# Supplementary figures and images for: Networks of care for optimizing Primary Health Care Service Delivery in Ethiopia: Enhancing relational linkages and care coordination
Source: PLoS One. 2025 Jan 3;20(1):e0314807. doi: 10.1371/journal.pone.0314807 (PMC11698449; doi:10.1371/journal.pone.0314807)

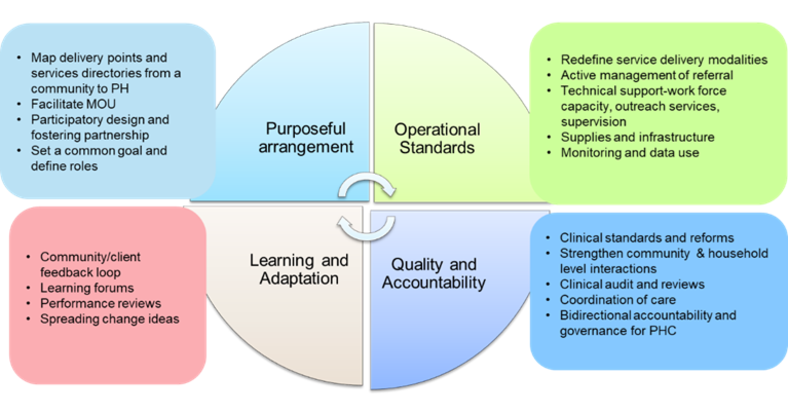

Supplement: S1 Fig — (TIF) [file pone.0314807.s001.tif]

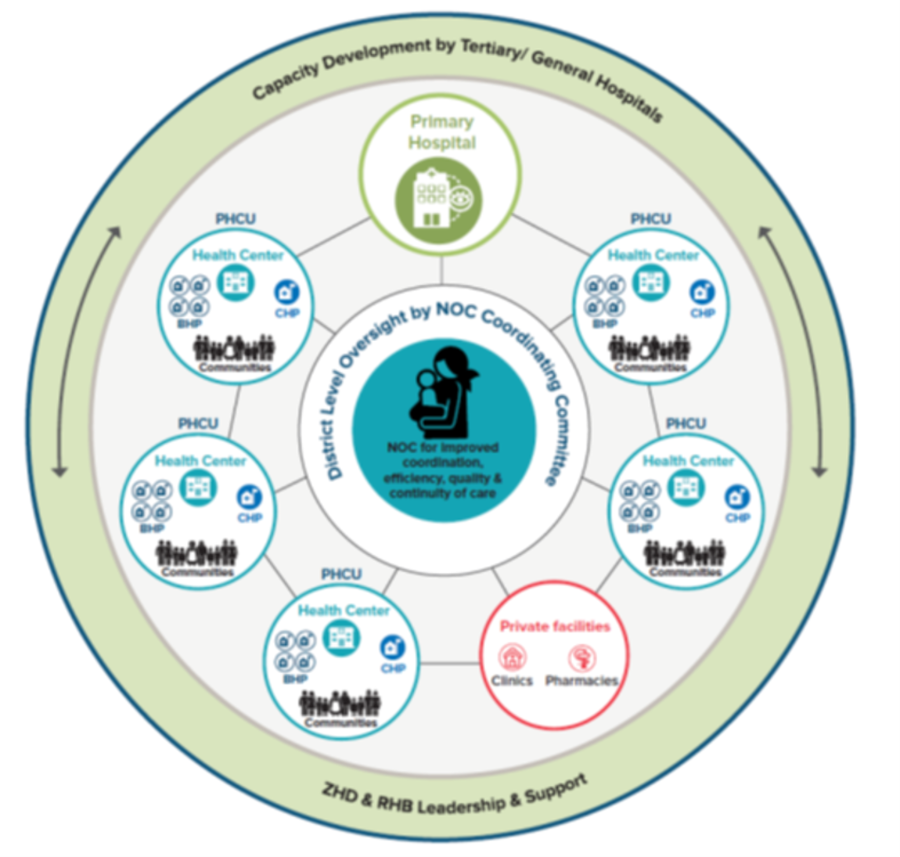

Supplement: S2 Fig — (TIF) [file pone.0314807.s002.tif]

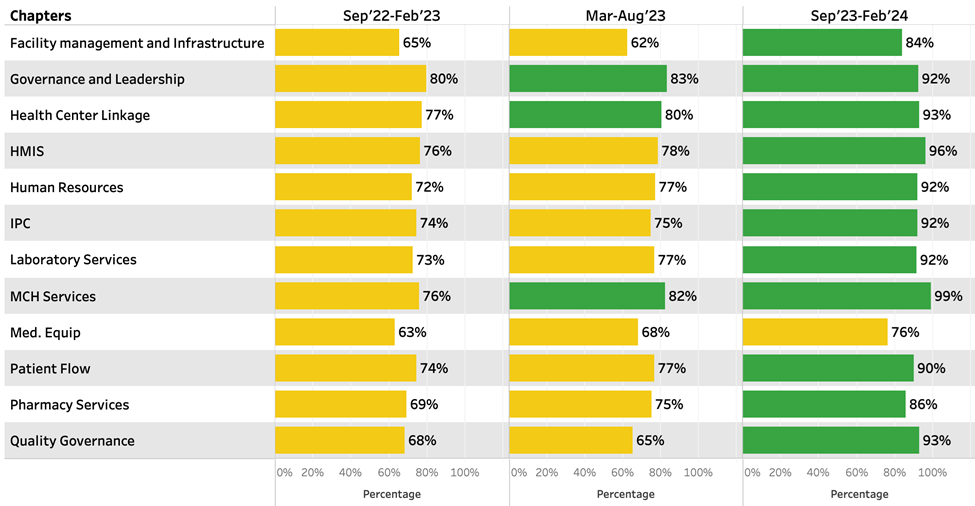

Supplement: S3 Fig — (TIF) [file pone.0314807.s003.tif]
