# Supplementary material for: Networks of care for optimizing Primary Health Care Service Delivery in Ethiopia: Enhancing relational linkages and care coordination
Source: PLoS One. 2025 Jan 3;20(1):e0314807. doi: 10.1371/journal.pone.0314807 (PMC11698449; doi:10.1371/journal.pone.0314807)
Supplement: S1 Table — (DOCX) [file pone.0314807.s004.docx]

*S3 Table: Roles and responsibilities of each level within the NOC framework*

| **Functions** | **Community/health posts** | **Health center/Clinics** | **Primary hospitals** | **Woreda health office** |
| --- | --- | --- | --- | --- |
| **Purposeful Arrangement** | - Participated in design and NOC establishment workshop  - Set health post-level goals aligned with woreda-level goals | - Participated in design and NOC establishment workshop  - Set primary health care unit (PHCU)-level goals aligned with woreda-level goals | - Set hospital-level goals aligned with woreda-level goals | - Coordinate participatory engagement of member entities |
| **Operational Standards** | - Received support and resources  - Implement service standards (HEP reform and clinical standards)  - Enhance communication and referral systems  - Implement service standards reform and clinical standards) | - Implement service standards, communication, and feedback mechanisms  - Standardized communication and two-way referral systems | - Implement service standards, communication, and feedback mechanisms  - Standardized communication and two-way referral systems | - Technical oversight and coordination |
| **Quality and Accountability** | - Early identification and referral  - Strengthen community engagement  - Quality improvement process | - Quality MNH services including basic emergency obstetric and newborn care (BEmONC)  - Mentorship to HEWs  - Multidisciplinary team outreach services  - Quality improvement process | - emergency obstetric and newborn care (EmONC)  - Mentorship to health centers  - Quality improvement process | - Technical oversight and coordination |
| **Learning and Adaptation** | - Participated in the NOC coordinating committee  - Kebele-cabinet/steering committee meetings and reviews | - Participated in the NOC coordinating committee  - Facilitated PHCU-level reviews and NOC learning sessions | - Participated in the NOC coordinating committee  - Facilitate collaborative learning forums | - Performance reviews |
