# Supplementary material for: Networks of care for optimizing Primary Health Care Service Delivery in Ethiopia: Enhancing relational linkages and care coordination
Source: PLoS One. 2025 Jan 3;20(1):e0314807. doi: 10.1371/journal.pone.0314807 (PMC11698449; doi:10.1371/journal.pone.0314807)
Supplement: S1 File — This is a key informant interview guide we used to interview the participants in our study. (DOCX) [file pone.0314807.s005.docx]

# Key informant interview guide for NOC implementation

**Setting the stage**

1. How do you explain NOC? How is different from the existing referral and technical linkages of PHCUs?

**Program design and implementation**

1. How is the NOC strategy designed and being implemented? **Probe**: Was it participatory and contextualized to your local situations? Was the common goal explicitly set and the role and responsibilities of each member facilities defined?
2. How do you evaluate the overall implementation of NOC? **Probe** for care coordination among the NOC member facilities including the community, bi-directional referral linkages, communication, and resource sharing. What worked well? Why? And why not?

**Perceived impact of the initiative**

1. Explain to us any changes you observed as the result of the implementation of the NOC strategy. **Probe** for;
   1. how NOC impacted the operationalization of the HEP roadmap and PHC service delivery,
   2. referral management, effective care coordination, and facility linkages across the PHC continuum of care,
   3. the impact on client experience of continuity of care and clients navigation of the health system
   4. trustful collaboration across levels of providers and facilities,
   5. technical and administrative oversight including resource sharing to fulfill supply and infrastructure shortages
   6. adherence to clinical and administrative standards,
   7. data-informed continuous quality improvement, and
   8. targeted skills building through in-service learning and clinical mentorship.
   9. Any unintended consequences or negative impact of the NOC strategy brought to your facility
2. What are the particular features of the NOC that made a difference?

**Monitoring and support system**

1. How did you monitor and evaluate the NOC implementation? **Probe**: Presence of quality structure or coordinating committee, supportive supervision, review meetings, or collaborative learning platforms
2. What specific support from the NOC member facilities did your facility receive? **Probe** for technical and managerial support

**Barriers and facilitators**

1. What are the facilitators of implementing NOC to optimize HEP and PHC service delivery as well as efficient and effective care coordination for improved RMNCH services? What factors affect the effectiveness of bidirectional linkages and technical and administrative support?
2. What are the hindrances to implementing NOC to achieve these objectives and outcomes?

**Areas of improvement**

1. How can the strategy be better implemented so that it could be more effective in achieving efficient service delivery and client-centered care provision?
