# Supplementary material for: Networks of care for optimizing Primary Health Care Service Delivery in Ethiopia: Enhancing relational linkages and care coordination
Source: PLoS One. 2025 Jan 3;20(1):e0314807. doi: 10.1371/journal.pone.0314807 (PMC11698449; doi:10.1371/journal.pone.0314807)
Supplement: Attachment — Submitted filename: Response to Reviewers.docx [file pone.0314807.s007.docx]

A point-by-point response to reviews

Dear Editor,

We, the authors, would like to express our gratitude to the reviewers and editors of PLOS ONE Journal for their valuable comments, which we believe have significantly strengthened our paper. Below, we provide point-by-point responses to each comments and suggestions. We have also reviewed the manuscript to ensure that it conforms to the journal's style.

**Journal Requirements:**

When submitting your revision, we need you to address these additional requirements.

1. Please ensure that your manuscript meets PLOS ONE's style requirements, including those for file naming. The PLOS ONE style templates can be found at

https://journals.plos.org/plosone/s/file?id=wjVg/PLOSOne_formatting_sample_main_body.pdf and

https://journals.plos.org/plosone/s/file?id=ba62/PLOSOne_formatting_sample_title_authors_affiliations.pdf.

Response: We reviewed to ensure that this version of the manuscript conforms to the journal style.

2. Please provide additional details regarding participant consent. In the ethics statement in the Methods and online submission information, please ensure that you have specified (1) whether consent was informed and (2) what type you obtained (for instance, written or verbal, and if verbal, how it was documented and witnessed). If your study included minors, state whether you obtained consent from parents or guardians. If the need for consent was waived by the ethics committee, please include this information.

If you are reporting a retrospective study of medical records or archived samples, please ensure that you have discussed whether all data were fully anonymized before you accessed them and/or whether the IRB or ethics committee waived the requirement for informed consent. If patients provided informed written consent to have data from their medical records used in research, please include this information.

Response: Thank you. The ethics statement has been revised to include details on consent. The following sentences are now part of the revised version (page 12 lines 214-220):

*“For the retrospective service statistics from medical records, data were collected in monthly aggregates and fully anonymized prior to access. The IRB waived the requirement for client informed consent; however, we obtained consent from facility directors. Informed written consent was also secured from key informants for qualitative interviews. All information obtained from informants was de-identified before analysis, and we maintained strict confidentiality throughout all stages of the study. “*

3. Thank you for stating the following in the Acknowledgments Section of your manuscript:

“We would like to thank the Bill & Melinda Gates Foundation for their generous funding and guidance throughout the project design and implementation”

We note that you have provided additional information within the Acknowledgements Section that is not currently declared in your Funding Statement. Please note that funding information should not appear in the Acknowledgments section or other areas of your manuscript. We will only publish funding information present in the Funding Statement section of the online submission form.

Please remove any funding-related text from the manuscript and let us know how you would like to update your Funding Statement. Currently, your Funding Statement reads as follows:

“The project is funded by the Bill & Melinda Gates Foundation and implemented through a collaborative consortium of Amref Health Africa and JSI. The funder has no role in the interpretation and implications of the content in this paper. This responsibility lies solely with the authors.”

Please include your amended statements within your cover letter; we will change the online submission form on your behalf.

Response: Thanks. We have removed the funding-related text from the manuscript. The funding statement contains all necessary declarations and does not require any updates.

4. We note that your Data Availability Statement is currently as follows: [All data used for this analysis is included in this report.]

Please confirm at this time whether or not your submission contains all raw data required to replicate the results of your study. Authors must share the “minimal data set” for their submission. PLOS defines the minimal data set to consist of the data required to replicate all study findings reported in the article, as well as related metadata and methods (https://journals.plos.org/plosone/s/data-availability#loc-minimal-data-set-definition).

For example, authors should submit the following data:

- The values behind the means, standard deviations and other measures reported;

- The values used to build graphs;

- The points extracted from images for analysis.

Authors do not need to submit their entire data set if only a portion of the data was used in the reported study.

If your submission does not contain these data, please either upload them as Supporting Information files or deposit them to a stable, public repository and provide us with the relevant URLs, DOIs, or accession numbers. For a list of recommended repositories, please see https://journals.plos.org/plosone/s/recommended-repositories.

If there are ethical or legal restrictions on sharing a de-identified data set, please explain them in detail (e.g., data contain potentially sensitive information, data are owned by a third-party organization, etc.) and who has imposed them (e.g., an ethics committee). Please also provide contact information for a data access committee, ethics committee, or other institutional body to which data requests may be sent. If data are owned by a third party, please indicate how others may request data access.

Response: The qualitative KII guide and the data used for this analysis have been included as Supplementary Information in S4 and S6, respectively.

5. We note that you have included the phrase “data not shown” in your manuscript. Unfortunately, this does not meet our data sharing requirements. PLOS does not permit references to inaccessible data. We require that authors provide all relevant data within the paper, Supporting Information files, or in an acceptable, public repository. Please add a citation to support this phrase or upload the data that corresponds with these findings to a stable repository (such as Fig share or Dryad) and provide and URLs, DOIs, or accession numbers that may be used to access these data. Or, if the data are not a core part of the research being presented in your study, we ask that you remove the phrase that refers to these data.

Response: Thanks. Figure 5 is provided instead (See page 21, lines 408-409)

**Review Comments to the Author**

Reviewer #1: The article is comprehensive and provides detailed insights into the topic, but some sections could be streamlined for clarity.

1. The section detailing the Networks of Care (NOC) is overly long and could benefit from conciseness. Summarizing key elements of NOC—such as its role in improving coordination, resource sharing, and maternal care outcomes—would make the manuscript more accessible without losing important information.

Response: Thank you for your valuable comment. In this version, we have significantly trimmed this section without losing key information and added S2 Table as supplementary information.

2. The mention of the RE-AIM framework feels underdeveloped. If the authors are not applying it fully by reporting on key dimensions like reach, adoption, implementation, and maintenance, it may be better to omit the framework from the methodology or expand its use and provide relevant data for each component.

Response: Comment acknowledged. Since we did not report on all dimensions of the RE-AIM framework, we have removed it in this version.

3. Fidelity should be clearly defined. Currently, it is discussed without specifying what components of the intervention were monitored for adherence, or how deviations were measured. A more structured discussion on how fidelity was evaluated would improve the rigor of the paper.

Response: Comment acknowledged. In the measurement section, we have now defined fidelity and outlined how we measured it, see Table 1 (page 10, line 188)

4. A summary table showing key indicators and the corresponding data collection methods would be helpful for readers. This would clarify how the study measured outcomes and tracked progress over time.

Response: Comment well taken and Table 1 presents the key indicators measured, along with their definitions and data sources (page 10, line 183)

5. The key informant interview methodology was very briefly mentioned ("Qualitative data from key informant interviews were transcribed, coded, and thematically analyzed using Atlas.ti"). I suggest following a standard checklist, such as COREQ, to provide more detailed information on qualitative data collection, including sampling, interview guides, and the coding process.

Response: Details of the KII data collection, the tool used (included as S4), and the analysis are now included.

The following paragraph is included in the data collection section (See page 9, lines 177-184).

*“Qualitative data were collected in March 2024 by the project M&E and program managers using an interview guide with open-ended and probing questions to gather insights from program implementers. The main topics of discussion included the differences between NOC and existing linkages, participatory and contextualized design, roles and responsibilities of network members, care coordination and communication, operational impact on PHC and HEP, collaboration and oversight, adherence to standards and quality improvement, monitoring and evaluation mechanisms, support systems and resource sharing, barriers and facilitators, unintended consequences, and areas for improvement (S6 File). Interviews were conducted at convenient locations within the participants' work environment.”*

The Analysis section is also revised as follows (see page 12, lines 202-207);

*“All interviews were audio-taped with the consent of the study participants and the records were transcribed verbatim by the project M&E team. The qualitative data from KIIs were transcribed, coded, and thematically analyzed using Atlas.ti. Priori codes were generated from the interview guide. Reports of quotations, themes, and patterns of interrelationships between the themes were identified. and quantitative data were integrated during both the presentation and interpretation stages.”*
