# Supplementary material for: Networks of care for optimizing Primary Health Care Service Delivery in Ethiopia: Enhancing relational linkages and care coordination
Source: PLoS One. 2025 Jan 3;20(1):e0314807. doi: 10.1371/journal.pone.0314807 (PMC11698449; doi:10.1371/journal.pone.0314807)
Supplement: Attachment — Submitted filename: review NOC.docx [file pone.0314807.s008.docx]

Thank you for the revision. The manuscript has improved in many ways. However, I have included my responses and a few additional comments below (in purple).

Overall comments

Previous responses and comments

1. Thank you for the revision. Table S2 provides an adequate overview of the framework.

2. The mention of the RE-AIM framework feels underdeveloped. If the authors are not

applying it fully by reporting on key dimensions like reach, adoption, implementation,

and maintenance, it may be better to omit the framework from the methodology or

expand its use and provide relevant data for each component.

Response: Comment acknowledged. Since we did not report on all dimensions of the

RE-AIM framework, we have removed it in this version.

In my opinion, if the authors do not expand on their use of the RE-AIM framework (specifying which components guided which indicators and acknowledging any components omitted due to limitations), they should consider mentioning other frameworks that may better suit the manuscript. Otherwise, readers may be confused about how each indicator was selected for analysis.

3. Fidelity should be clearly defined. Currently, it is discussed without specifying what

components of the intervention were monitored for adherence, or how deviations were

measured. A more structured discussion on how fidelity was evaluated would improve

the rigor of the paper.

Response: Comment acknowledged. In the measurement section, we have now

defined fidelity and outlined how we measured it, see Table 1 (page 10, line 188)

Thank you for defining fidelity. However, in line with the previous comment, the presentation of other indicators is confusing, as they don’t seem to clearly adhere to any specific framework.

For example, in the results section of the main text, the authors briefly mention fidelity with the statement: “**Fidelity**: A sizable progress in NoCs implementation fidelity was achieved.” Then, under the Fidelity subsection, functionality is reported instead: “The NoCs functionality score improved from 56% to 89% in agrarian settings and from 38% to 60% in pastoral settings (Fig 2). Improvements included enhanced coordination, standardized referral practices, and increased resource sharing among facilities. Coaching and mentorship strengthened at primary care levels, improving efficiency, access, and quality of care.”

This is confusing because the authors previously defined functionality as a separate indicator. Following a specific implementation evaluation framework (such as the RE-AIM framework or the MRC Process Evaluation Framework, <https://pubmed.ncbi.nlm.nih.gov/25791983/>) might clarify the structure and interpretation.

4. “A summary table showing key indicators and the corresponding data collection

methods would be helpful for readers. This would clarify how the study measured

outcomes and tracked progress over time.

Response: Comment well taken and Table 1 presents the key indicators measured,

along with their definitions and data sources (page 10, line 183)”:

See comments above.

Additional comments

1. Please avoid overusing abbreviations, as it can make the text difficult to understand. For example, "QI" should be written in full.
2. Results (abstract)

*“Results: The NoCs approach has significantly strengthened both administrative and technical connections, while enhancing relational linkages, multi-level collaboration, and bidirectional communication. This has fostered trust, improved care coordination, boosted primary health care performance, and increased health service utilization within woreda health systems. The Interrupted time series analysis indicated that the rate of ANC 8+ visits was 29.8% per month higher than expected without the NoCs strategy (Coef: 2.39; p-value < 0.01) and an 18.4% increase in obstetric complications managed (Coef: 1.71; p-value 36 = 0.050), with a 43% overall increase. Perinatal mortality decreased by 34%, from 31.3 to 20.1 per 1,000 births [t-test: 2.12; p-value: 0.040)].”*

Could you clarify the definitions and distinctions between “administrative connections,” “technical connections,” “relational linkages,” “multi-level collaboration,” and “bidirectional communication”?

The results section in the main text reports different indicators than these terms. Could you explain the reason for this discrepancy? Furthermore, why does the abstract use a different format, such as “Implementation fidelity, challenges, and adaptations,” “Enhanced bidirectional linkages,” “Optimized HEP and improved performance and efficiency of the PHC system,” and “Improved utilization, quality, and continuity of care”?

3. Discussions

*“…Previous studies underscore that involving communities in these networks and quality improvement efforts significantly boosts maternal health program effectiveness. By engaging community members, local leaders, and stakeholders in service design, implementation, and evaluation, programs become more accessible, culturally sensitive, and widely utilized. Initiatives like participatory learning groups, community health committees, and training for health workers increase antenatal visits, skilled birth attendance, and postnatal care, ultimately lowering maternal and neonatal mortality rates [22-24]. These engaged networks build trust between health care providers and communities, ensuring interventions meet local needs sustainably.”*

This section would benefit from additional references. For example, the statement “…Previous studies underscore that involving communities in these networks and quality improvement efforts significantly boosts maternal health program effectiveness” should be followed by citations that demonstrate the effectiveness of “involving communities in these networks…”

Additionally, the discussion section appears to emphasize the importance of the NOC framework without comparing other aspects of the research, such as fidelity, functionality, or the evaluation methodology, with existing literature. Please consider including these comparisons.

4. Please provide the study’s limitations.
5. Please provide necessary information about the shared data in Supplementary #6.csv (e.g., brief descriptions, data dictionary).
