# Supplementary material for: Networks of care for optimizing Primary Health Care Service Delivery in Ethiopia: Enhancing relational linkages and care coordination
Source: PLoS One. 2025 Jan 3;20(1):e0314807. doi: 10.1371/journal.pone.0314807 (PMC11698449; doi:10.1371/journal.pone.0314807)
Supplement: Attachment — Submitted filename: Response to Reviewers.docx [file pone.0314807.s009.docx]

A point-by-point response to reviews

Dear Editor,

We, the authors, would like to express our gratitude to the reviewers and editors of PLOS ONE Journal for their valuable comments, which we believe have significantly strengthened our paper. Below, we provide point-by-point responses to each comment and suggestions in red font. We have also reviewed the manuscript to ensure that it conforms to the journal's style.

**Editor comment**

The manuscript has been implemented compared to the first version. However, you must complete the review in accordance with the guidance provided by the reviewers. Furthermore, it is necessary to explain the changes that have occurred in the list of authors, since a new author has been added and to certify that all the authors of the manuscript are aware of and in agreement with them, in compliance with the policy of the Journal PLOS ONE.

Response: Thank you. We have carefully addressed all the feedback provided by the reviewer in this version. Additionally, including a new author has been communicated to all contributors. We inadvertently omitted his name from the author list in the initial submission. However, he contributed substantially to the manuscript, including project design, data acquisition, conceptualization, and review.

**Journal Requirements:**

Please review your reference list to ensure that it is complete and correct. If you have cited papers that have been retracted, please include the rationale for doing so in the manuscript text, or remove these references and replace them with relevant current references. Any changes to the reference list should be mentioned in the rebuttal letter that accompanies your revised manuscript. If you need to cite a retracted article, indicate the article’s retracted status in the References list and also include a citation and full reference for the retraction notice.

Response: The reference lists have been reviewed, and minor corrections have been made to the format

**Reviewers' comments:**

Reviewer #1: Thank you for the revision. The manuscript has improved in many ways. However, I have included my responses and a few additional comments below (in purple).

Response: Thank you for the valuable comments, which have been essential for enhancing the scientific rigor of this manuscript. We have carefully addressed all feedback in this version.

Overall comments

Previous responses and comments

1. Thank you for the revision. Table S2 provides an adequate overview of the framework.

Response: Thank you.

2. "The mention of the RE-AIM framework feels underdeveloped. If the authors are not applying it fully by reporting on key dimensions like reach, adoption, implementation, and maintenance, it may be better to omit the framework from the methodology or expand its use and provide relevant data for each component.

Response: Comment acknowledged. Since we did not report on all dimensions of the RE-AIM framework, we have removed it in this version."

Further comments:

In my opinion, if the authors do not expand on their use of the RE-AIM framework (specifying which components guided which indicators and acknowledging any components omitted due to limitations), they should consider mentioning other frameworks that may better suit the manuscript. Otherwise, readers may be confused about how each indicator was selected for analysis.

Response: Thank you again for the valid and valuable feedback. In this version, Table 1 has been substantially revised, with indicators mapped to the RE-AIM outcomes. Some outcome measures, such as Maintenance (sustainability), are not adequately measured and are addressed in the limitations section of the manuscript. **See Pages 8-9, lines 157-159, and Table 1 pages 10-11, lines 193-197 as well as page 14, lines 230-234 on Revised Manuscript with Track Changes.**

3. Fidelity should be clearly defined. Currently, it is discussed without specifying what

components of the intervention were monitored for adherence, or how deviations were

measured. A more structured discussion on how fidelity was evaluated would improve

the rigor of the paper.

Response: Comment acknowledged. In the measurement section, we have now

defined fidelity and outlined how we measured it, see Table 1 (page 10, line 188)

Further comments:

Thank you for defining fidelity. However, in line with the previous comment, the presentation of other indicators is confusing, as they don’t seem to clearly adhere to any specific framework.

For example, in the results section of the main text, the authors briefly mention fidelity with the statement: “**Fidelity**: A sizable progress in NoCs implementation fidelity was achieved.” Then, under the Fidelity subsection, functionality is reported instead: “The NoCs functionality score improved from 56% to 89% in agrarian settings and from 38% to 60% in pastoral settings (Fig 2). Improvements included enhanced coordination, standardized referral practices, and increased resource sharing among facilities. Coaching and mentorship strengthened at primary care levels, improving efficiency, access, and quality of care.”

This is confusing because the authors previously defined functionality as a separate indicator. Following a specific implementation evaluation framework (such as the RE-AIM framework or the MRC Process Evaluation Framework, <https://pubmed.ncbi.nlm.nih.gov/25791983/>) might clarify the structure and interpretation.

Response: As described above, in this version, fidelity is measured and defined by NoC functionality and maturity metrics, as well as by contextual challenges and adaptations in alignment with the RE-AIM framework. **Table 1 pages 10-11, lines 193-197 on Revised Manuscript with Track Changes**

4. “A summary table showing key indicators and the corresponding data collection

methods would be helpful for readers. This would clarify how the study measured

outcomes and tracked progress over time.

Response: Comment well taken and Table 1 presents the key indicators measured,

along with their definitions and data sources (page 10, line 183)”.

Further comments: See comments above.

Response: The indicators measured are presented in the revised Table 1, along with their definitions and data sources, in alignment with the RE-AIM framework. **Table 1 pages 10-11, lines 193-197 on Revised Manuscript with Track Changes**

Additional comments

1. Please avoid overusing abbreviations, as it can make the text difficult to understand. For example, "QI" should be written in full.

Response: Thank you for bringing this to our attention. In this version, we have removed abbreviations that are not frequently used in the text and written them out in full, including terms like QI, PDSA, FRAME-IS, ERIC, WDA, MOU, and EHCRIG.

2. Results (abstract)

“Results: The NoCs approach has significantly strengthened both administrative and technical connections, while enhancing relational linkages, multi-level collaboration, and bidirectional communication. This has fostered trust, improved care coordination, boosted primary health care performance, and increased health service utilization within woreda health systems. The Interrupted time series analysis indicated that the rate of ANC 8+ visits was 29.8% per month higher than expected without the NoCs strategy (Coef: 2.39; p-value < 0.01) and an 18.4% increase in obstetric complications managed (Coef: 1.71; p-value 36 = 0.050), with a 43% overall increase. Perinatal mortality decreased by 34%, from 31.3 to 20.1 per 1,000 births [t-test: 2.12; p-value: 0.040)].”

Could you clarify the definitions and distinctions between “administrative connections,” “technical connections,” “relational linkages,” “multi-level collaboration,” and “bidirectional communication”?

The results section in the main text reports different indicators than these terms. Could you explain the reason for this discrepancy? Furthermore, why does the abstract use a different format, such as “Implementation fidelity, challenges, and adaptations,” “Enhanced bidirectional linkages,” “Optimized HEP and improved performance and efficiency of the PHC system,” and “Improved utilization, quality, and continuity of care”?

Response: Thank you for the comments. We have revised the abstract (Results) section to provide greater clarity for readers as shown below.

*“The NoCs approach has significantly enhanced relational linkages between primary health care facilities and health care providers, fostering stronger collaboration and communication. This has fostered trust, improved care coordination, optimized primary health care performance, and increased health service utilization within woreda health systems. The Interrupted time series analysis indicated that the rate of ANC 8+ visits was 29.8% per month higher than expected without the NoCs strategy (Coef: 2.39; p-value < 0.01) and an 18.4% increase in obstetric complications managed (Coef: 1.71; p-value = 0.050), with a 43% overall increase. Perinatal mortality decreased by 34%, from 31.3 to 20.1 per 1,000 births [t-test: 2.12; p-value: 0.040)].”* **See Pages 2-3, lines 31-40 on Revised Manuscript with Track Changes.**

3. Discussions

“…Previous studies underscore that involving communities in these networks and quality improvement efforts significantly boosts maternal health program effectiveness. By engaging community members, local leaders, and stakeholders in service design, implementation, and evaluation, programs become more accessible, culturally sensitive, and widely utilized. Initiatives like participatory learning groups, community health committees, and training for health workers increase antenatal visits, skilled birth attendance, and postnatal care, ultimately lowering maternal and neonatal mortality rates [22-24]. These engaged networks build trust between health care providers and communities, ensuring interventions meet local needs sustainably.”

This section would benefit from additional references. For example, the statement “…Previous studies underscore that involving communities in these networks and quality improvement efforts significantly boosts maternal health program effectiveness” should be followed by citations that demonstrate the effectiveness of “involving communities in these networks…”

Response: Thank you 3 references are now included as suggested. **Page 25, line 458 on Revised Manuscript with Track Changes**

Additionally, the discussion section appears to emphasize the importance of the NOC framework without comparing other aspects of the research, such as fidelity, functionality, or the evaluation methodology, with existing literature. Please consider including these comparisons.

Response: Thank you for your valuable comments. In this version, we compared the use of implementation science research methodologies, bridging the knowledge gap left by previous studies focused on clinical experiences and case study descriptions. This study explored the mechanisms shaping networks between PHC facilities and communities. Additionally, we discussed how fidelity and adaptation influence implementation outcomes and addressed the limitations caused by inadequate fidelity and program implementation, particularly in assessing RE-AIM outcomes like Maintenance (sustainability). **Page 26 lines 493-495; *Page 27, lines 497-498; and lines 507-513* on Revised Manuscript with Track Changes**

4. Please provide the study’s limitations.

Response: Thank you for the valid comment. The following paragraph is included in this version.

*"Adapting programs to local contexts is crucial for ensuring strategies are both practical and effective. High-fidelity implementation improves PHC performance, health outcomes, and sustainability. However, due to the study’s limited duration, certain RE-AIM measures, such as sustainability (Maintenance), were not fully assessed, limiting the evaluation of NoCs’ maturity and integration into health system processes. Additionally, the absence of a comparison group may weaken the evidence from the time-series analysis, and the functionality and maturity matrix could be influenced by assessor bias and implementation variability."* ***Page 27, lines 507-513* on Revised Manuscript with Track Changes**

5. Please provide necessary information about the shared data in Supplementary #6.csv (e.g., brief descriptions, data dictionary).

Response: Apologies for overlooking the data dictionary. In this version, a data dictionary and brief description have been included as separate sheets labeled 'Data Dictionary' and 'Description.'
